# Family Food Environment during the COVID-19 Pandemic: A Qualitative Study

**DOI:** 10.3390/children8050354

**Published:** 2021-04-29

**Authors:** Amber J. Hammons, Ryan Robart

**Affiliations:** Department of Child and Family Science, California State University, Fresno, CA 93740, USA; rrobart@csufresno.edu

**Keywords:** family food environment, family mealtimes, family functioning, COVID-19 pandemic

## Abstract

Background: The COVID-19 pandemic, with its cyclical lockdown restrictions and school closures, has influenced family life. The home, work, and school environments have collided and merged to form a new normal for many families. This merging extends into the family food environment, and little is known about how families are currently navigating this landscape. The objective of the present study was to describe families’ adaptations in the family food environment during the COVID-19 pandemic. Methods: Parents participated in one of 14 virtual focus groups (conducted in English and Spanish between December 2020 and February 2021). Reflexive thematic analysis was used to analyze the transcripts. Results: Forty-eight parents (81% Hispanic and SES diverse) participated. Five themes and one subtheme were identified around changes in eating habits and mealtime frequency, increases in snacking, family connectedness at mealtimes, and use of screens at meals. Conclusions: The COVID-19 pandemic has influenced the family food environment. Families shared how their eating habits have changed and that device usage increased at mealtimes. Some changes (e.g., weight gain) may have lasting health implications for both children and parents. Public health officials, pediatricians, and schools should work with families to resume healthy habits post pandemic.

## 1. Introduction

The COVID-19 pandemic has forced families to restructure their daily lives. Cyclical lockdown restrictions, school closures, remote work, and closures of family-oriented recreation have caused a collapse of normal routines. Children and adults are spending more time at home and consuming food and meals that would have otherwise taken place in different settings. There is strong support for the role that parents play in shaping children’s eating habits, especially through provision of specific types of foods, accessibility [1], involvement [2,3,4,5], and modeling [6,7,8], which all may be affected during a pandemic. The COVID-19 pandemic is likely to serve as an impetus for changes in the family food environment.

In times of uncertainty, family routines, such as mealtimes, may help families maintain a semblance of normalcy. Family mealtimes may be protective [9], providing families a way to feel connected [10,11,12], united, and secure during times of stress. A recent meta-analysis found that family mealtime frequency is related to better family functioning, which includes problem-solving, communication, and feelings of family connectedness [13]. Additionally, family meals provide a central place where family members come together and where healthy eating habits can be promoted. The frequency of shared family mealtimes has been associated with many benefits including healthy eating [14], especially fruits and vegetables [15], and overall diet quality [16].

Mealtime structure variables, which include eating together as a family, device usage, and types of foods consumed, directly influence children’s eating patterns [17]. During times of stress, the family food environment is susceptible to changes in healthy behaviors [18]. Structured meals with positive interpersonal dynamics have been associated with higher levels of vegetable consumption and lower BMIs [19], while poor communication, lower interpersonal involvement, and negativity have been associated with less healthy eating patterns [5,20]. Mealtime stress induced using an experimental design was associated with greater consumption of sweets and lower levels of positive social interaction in families [21]. In one study, women that experienced high stress consumed more food after stressful events [22]. Mealtimes are but one aspect of the family food environment. Shopping for food, cooking, and snacking also comprise the family food environment and are likely to be altered during chronic stressful periods.

According to the Family Adjustment and Adaptation Response Model, when families experience stressors that exceed their coping capabilities, initiating a crisis, families will adapt in the direction of either enhanced functioning or poor functioning [23]. All families are currently experiencing the global stressor of a pandemic but the ways in which lives have been disrupted for individual families varies in part on the basis of resources and family capabilities. For many families, the pandemic has meant spending inordinate amounts of time together, in very close proximity, within the home. In recent research, families reported higher levels of stress during the COVID-19 pandemic compared to pre pandemic [24], and eating behaviors included increased consumption of junk food during the first 3 months [18]. Similarly, other studies using survey research during the first couple of months of the pandemic also found changes in eating habits [25] (e.g., increased processed foods, snack foods [26], and fresh foods in the home [27]), as well as changes in parent feeding practices, such as increases in restriction, monitoring, and pressure to eat [27].

During times of uncertainty, family functioning may change as families attempt to cope with the situation. Due to the unprecedented nature of a global pandemic, there is little research on how families are navigating this new family landscape. This is an exploratory study examining how families have adjusted during the COVID-19 pandemic, specifically in relation to the family food environment and family mealtimes. This research is important because it can potentially inform family wellness efforts to help families as they transition to a new normal.

## 2. Materials and Methods

Parents participated in a virtual focus group session via Zoom about family functioning during the COVID-19 pandemic. Focus group sessions took place between December 2020 and February 2021. This study is part of a qualitative study on family functioning during the COVID-19 pandemic and was approved by the Department of Child and Family Science’s Review Committee for the Protection of Human Subjects at California State University, Fresno (Protocol code # 0002, 11/24/20).

### 2.1. Participants and Recruitment

Eligibility criteria included being a parent of a child between the ages of 5 and 18 and having access to the internet. Parents were recruited through a previous contact list (pre pandemic) of families that indicated an interest in receiving more information about participating in family-based research for Hispanic families. Parents were also recruited through announcements made on social media, an email sent to a student listserv in the Child and Family Science Department at the university, snowballing, and word of mouth. Parents participated in one of 14 focus groups, which were offered in English (*n* = 6) and Spanish (*n* = 8). A total of 48 parents, (47 mothers, one father) living in California participated. An average of three parents participated in each group. The sample was 81% Hispanic, with an average parent age of 37.48 (SD = 8.37). Parents had an average of three children, with an average child sample age of 11.13 (SD = 5.87). Fifty-eight percent of parents had a high-school degree or less. Seventy-nine percent were married or cohabiting, and 60% of the sample reported an annual income of less than $50,000. While 38% of the participants in the sample were currently employed, exactly half of them were working remotely. Fifty-two percent of the sample were essential workers or lived with an essential worker. All children were attending school remotely during the time of the study. See Table 1 for additional sample characteristics.

### 2.2. Measures

Demographic information. Demographic information included parent age, child ages, children’s schooling (remote or in-person), ethnicity, marital status, employment status, essential worker status, remote work, income, education, and vaccination interest.

Focus group guide. A semi-structured interview guide was developed by the research team on the basis of previous studies (e.g., see [28,29]) and a review of the literature on family process and family functioning. Family food environment and family mealtimes were areas of particular interest. Questions were open-ended. Sample questions included the following: “What does a ‘family meal’ look like in your house right now? Who is typically there? Describe what happens during family meals. Have your family mealtimes changed since the pandemic began? If so, how?”

### 2.3. Procedure

Focus groups were used in this study because they work well for exploratory research [30,31] and we wanted to provide a space for families to share their experiences through an open and in-depth discussion. Due to lockdowns and university restrictions, all focus groups were conducted and recorded via Zoom (Zoom Video Communications; videotelephony software). Focus groups were conducted by two trained members of the research team (A.J.H. and project coordinator, G.G.), with prior experience in focus group methodology. Focus groups lasted an average of 70 min and included filling out a 5 min demographic survey at the end. The research team met regularly after the focus groups to discuss and track patterns, so as to determine when data saturation was achieved.

### 2.4. Analysis

Focus groups were transcribed verbatim in the language they were conducted in. The Spanish transcriptions were then translated into English by three proficient bilingual Spanish/English research assistants. The English translation was then back-translated to Spanish by a research assistant with no exposure to the original transcription. Back-translations were compared to the original transcription for accuracy purposes. Disagreements were resolved by consensus. Thematic analysis [32], using an inductive approach and grounded in phenomenology, was applied to the transcripts. Study authors fully familiarized themselves with the transcripts before independently creating codes on the basis of patterns of meanings in the data. Codes were then discussed, agreed upon, and applied independently. Authors discussed the coding process and made refinements as necessary throughout. Themes were created on the basis of patterns of meanings, and representative quotes were selected and agreed upon by study authors. Participants were labeled by focus group number, the language the group was conducted in, and a random letter assigned to each person (e.g., 4SA = focus group #4, conducted in Spanish, person A). Data were organized and analyzed using the qualitative data analysis software program, Dedoose Version 8.3.47 [33].

## 3. Results

No differences were identified with regard to themes between groups conducted in English versus Spanish. Five themes and one subtheme were identified: (1) “We just eat a little more”: changes in eating habits; Subtheme: “We can see it in the size of the children’s clothing”: weight gain; (2) “It is constant, ‘Can I have a snack?’”: increases in snacking; (3) “We do it every day”: changes in mealtime frequency; (4) “It’s been very positive”: family mealtimes have helped keep family members connected during the pandemic; (5) “They just want to be glued to a tablet while they eat”: increase in screen time during meals.

### 3.1. “We Just Eat a Little More”: Changes in Eating Habits

The majority of parents shared that their family’s eating habits had changed in some way since the pandemic began. Some families found that the quantity of food consumed had changed, “more excess, more times, now they eat 6, 7, 8 times a day” (7SC), with larger portion sizes and more food overall each day. One mother shared, “they [children] try to eat more things because they are at home and have nothing to do” (2SG). For some, they found that the types of foods they purchased had changed, “the children sometimes get up and go directly to the refrigerator or at dinner to get some candy, to look for cookies and things that we hardly bought before now”, as well as the quantity, “I have to buy them more because they go and raid the refrigerator very often during the day” (5SO). The increase in time spent at home and the subsequent increase in food consumption strained financial resources for some, limiting what they could purchase and eat, “my problem is my kids are home so much more and so I would like to cook healthier, it is just way more expensive” (5EA). Another mother shared that, pre pandemic, the food situation was better because children received food at school, but that with children at home this was no longer the case, and “now I have to buy the cheapest, and well I have to buy what I can afford” (2SX). Several families remarked on how the lockdowns and closures of restaurants resulted in a lower consumption of eating out, especially less fast food, and that they were cooking more now as a result of “just being home all the time” (5ET). Similarly, another mother shared that, because they were home all day, she “spoils the kids more”, making “taquitos, browned chicken, pozole, mole”, and that they were “eating less healthy” (7SA). Some families aimed to eat healthier during the pandemic, “we are more intentional now about making sure that we have more nutritious meals more often” (6EB), but many of the families felt that overall they were mostly eating unhealthily during the pandemic. One mother described how her children’s food choices when shopping had changed since the pandemic began:

“Every time we go to the store, although sometimes I don’t take the girls, but the times I’ve taken them, I’ve noticed that they grab more things, like chips, like Hot Cheetos, sodas, things… just things that are not good for them. Cookies, cupcakes, things that I ask them why they grabbed all that, and they say, ‘I feel like it’. It’s the easiest thing they can get to take to the rooms to eat, and this wasn’t done before.”

(4SR)

While most families shared that their family’s eating habits had changed, some families shared that “it is more or less the same” (1EU) as pre pandemic. A few families shared how their regular feeding and shopping practices were habitual, for example, that they do not purchase unhealthy foods and, thus, were unaffected by the pandemic.

#### “We Can See It in the Size of the Children’s Clothing”: Weight Gain

Some parents talked about weight gain during the pandemic, as one mom remarked, “I knew that we were not the only family to notice that we were gaining weight” (7SO). Most parents attributed this to changes in eating habits, “snacking has definitely increased, and we can see it in the size of the children’s clothing” (5SO). They noticed changes in food consumption patterns, as well as corresponding changes in child and parent weight, “we have gained weight as I say, because we eat more” (7SC). A few parents also connected this to the increased sedentary behaviors, because “they’re [children] not exercising, they cannot be eating that much” (6S5), eating out of boredom, limited opportunities to do other things, and stress, “things are so stressful right now, I put on so much weight because of COVID” (2ET). One mom described what it has been like in her household:

“They finish their classes, we pick up, eat, and we watch TV, and it has to be Sabritas, there are some cookies, a cake, yes. Yes, the truth is, it hurts us to be in the house because we cannot go out to the park to run or play. We watch TV, we crave a bag of cookies, we bring them, or a bag of popcorn. The truth is, we are all gaining weight.”

(7S0)

### 3.2. “It Is Constant, ‘Can I Have a Snack?’”: Increases in Snacking

Most parents reported an increase in child snacking behaviors during the COVID-19 pandemic, as one mother shared, “I am 100% certain that their snacking is so much more than it would be if they were in school. There are no ifs, ands, or buts about it” (5EA). Parents attributed this to children being home more, increased accessibility to food in the pantry and kitchen, “just being home, before they didn’t have constant access to refrigerators and pantries” (3EQ), availability “and they know it’s there so they want to consume it more” (9SV), boredom, “they are not necessarily hungry because they just ate” (2EN), and increased stress. Some parents shared that their children would constantly ask to eat something, “she wants a snack all the time” (6S1), or regularly help themselves to food. For some, this became a struggle as parents found that available food would be consumed very “quickly, and then I have to buy more” (9SG), and that this made the food situation difficult to manage, “they will eat whatever is available and I think that is a problem” (5EA). While many parents shared that they provided fruits and vegetables as snacks, unhealthy snacks were also usually available and preferred, “if they don’t have sugar, they won’t eat it” (9SH), and “he will prefer the junk over the fruit” (3ER). Similarly, another mother shared:

“For their snack, pure fries are what they eat. If I make them, just like [other participant] said, something healthy, they don’t want that and they grab a sausage or a sandwich, that’s a snack for them. The way I see it, it’s like junk food.”

(2SX)

The majority of parents also shared that their own snacking had changed since the pandemic began, “I did increase a lot of snacks and, yes, I ate too much” (3SD), with most parents sharing that they were eating more, “it has changed for the bad” (2ED). Some parents linked their children’s snacking to an increase in their own snacking, “it’s easy to join in with the kids” (3ES). They would prepare snacks for their children and find themselves snacking as well, “I typically don’t like the type of snacks the boys like but if there’s some left or if there are extra ones I will tend to eat them just so they don’t go to waste” (4E4), and that this behavior was different from before the pandemic. Another parent shared that she “catches” the urge from her children to snack:

“There are even times that when they grab it, they offer it to me, and it makes it easier for me to say, ‘oh, yes’, or just to have a moment when we sit down to eat a snack together to talk. So, my consumption is also, it has increased a lot, especially because they tell me, ‘oh my partner ate such a snack. Will you buy it for me? My partner told me about such a snack, let’s try it.’ So, the increase has definitely increased for both of us.”

(5SV)

As the pandemic persisted, several parents shared that they have tried to transition from sweets and salty foods to more healthy snacks, or they have started to avoid purchasing unhealthy snacks altogether, “we also know when we go, what aisles to avoid to prevent us from bringing home those snacks” (2ED). Once one mother was able to return to work, her behavior changed, but she shared what it was like for her initially:

“I was snacking a lot, a whole lot of bad stuff because I don’t know, I would find myself going to the Dollar Store, at least I would walk to the Dollar Store. I would get my favorite candies, my chips, and I guess it was my way of coping, I guess. I was not working, I would get bored, and I would snack on all the bad stuff.”

(1E3)

### 3.3. “We Do It Every Day”: Changes in Mealtime Frequency

All families reported sharing a meal together with all family members present at least once a week. The majority of families experienced a change in the frequency of shared mealtimes during the pandemic, with most families sitting down together more often than pre pandemic. One parent shared:

“And so now we have meals like all together three times a day and, before the pandemic, we probably had dinner as a family together.”

(4EX)

Families were usually sharing lunch and dinner together daily; however, for some families, they were sharing only more dinners together because parents’ working situations had changed and this now meant that all family members would be together for the dinner meal.

### 3.4. “It’s Been Very Positive”: Family Mealtimes Have Helped Keep Family Members Connected during the Pandemic

Nearly all parents said that the additional time spent together during mealtimes has been positive for their families, “it has brought us together” (1S1), “I have my whole family at home” (7SA), and “anytime you can get more face time together is a good thing” (3EP). Parents discussed how it gave everyone an opportunity to connect, “we share more things, we can socialize more” (6SJ), and to check in to see how everyone was doing, “we understand each other better” (2SG) and “during the meal we can talk about fixing problems and things like that” (8SL). The increased time spent together also meant for many of the families that mealtime “is less chaotic and simpler” (6EF), resulting in a less rushed meal, “and now we have more time to talk” (6SA). Sometimes this meant that a parent who was working pre pandemic during this time or because “there’s no commute time” (6EF) could now be home for dinner, “my husband feels very happy that he gets to spend time with his family, it’s very nice that he’s actually there for dinner” (6EK) and that, pre pandemic, “it was rare to see that we sat together as a family” (3SD). One mother explained:

“I think it has been great. I think, you know, it is really hard with the job change. I feel like the kids are stuck home by themselves so much, my older kids especially. I feel a little more isolated, that we are not there as much. And, so, having the consistency of us sitting home and being together is like kind of a grounding focusing point for all of us. We are all there together, no one is missing. We are still a family; we are still here. Even though you may feel alone during the day, you know we are still functioning, we are still here. I think that has been good. It has given us a real sense of family and togetherness where a lot of times we were not having that because we were still working, there was no pandemic, the kids were going to school. Dinner time has given us a sense of normalcy.”

(5EA)

Part of what has made mealtimes so enjoyable for some families has been an increase in family member involvement in mealtimes, “I include them when it comes to eating, helping me, and even sometimes they give us ideas of what to eat” (6ST). The absence of organized activities and sports opened up time for families to spend together, especially around dinnertimes. For some families, this meant children helping more by setting the table and cleaning up, as one mother shared:

“For us, it has been more positive because my children have been learning, I have been delegating them responsibilities. Some set the table, others, when we finish, cleaning the table, others wash. We have begun to share that task because the majority were in class or work. We have bonded more and, for us, personally, it is very beneficial for me.”

(7SO)

In other families, it meant more family discussion about what types of foods would be served, “we have more time to talk and to decide what we want to eat, in general, we grab opinions from our children on what to eat” (3ST), and, in some families, it meant an increase in other family members cooking.

### 3.5. “They Just Want to Be Glued to a Tablet While They Eat”: Increase in Screen Time during Meals

The majority of families shared that the television and/or electronic devices were present at the meal table, “the phone, the iPad, the computer, or even the TV, video games, everything” (4SB). For many families, this represented an increase in device use compared to pre pandemic, “and now children want to have it [electronics] while they eat” (6SI), and that it was both parents and children that would have devices at the table, “sometimes like we’ll all be on our own devices to be honest, just like watching videos” (4EX). For some, this was just a continuance of device usage throughout the day, “let’s all face it, we are all addicted to technology in this life” (7SC). One mother shared:

“For the most part, something is always on. Even if something is just in the background, there is always some kind of noise going on. I would say we are not as great at keeping our phones off the table. Sometimes they are kind of distracting and I have to remind myself I have to put it down, it can wait, it is not that big of a deal. I personally reach for my phone way more than I should. It is just a habit. I am home all day. It is my way of socializing with the outside world. I sometimes forget that it can wait. My husband and my kids can use my undivided attention. So, the TV is usually on.”

(5ET)

With the exception of the TV, most parents were not happy about the use of devices at the table, “in my house, it’s honestly a fight all day; it even gets weird because my husband is on the phone” (4SB). While the majority of families reported using screens during mealtimes, some families shared that they had rules prohibiting their presence at the table and that this continued to be enforced throughout the pandemic, “my children do not have cell phones, the TV, everything has to be off, it is a rule they have had since they were boys” (3SA).

## 4. Discussion

The objective of this exploratory study was to describe how families have managed the family food environment during the COVID-19 pandemic. When the pandemic began, many parents found themselves straddling two worlds that typically do not directly collide in the home: work and children’s schooling. The merging of these settings also meant an overlap in eating and mealtimes that would have otherwise occurred elsewhere. Families scrambled to establish new food-related routines, while both healthy and unhealthy habits emerged. The present study revealed how the concomitant effects of the COVID-19 pandemic have influenced the family food environment. Families changed their eating behaviors, snacked more throughout the day, shared more meals together, and used electronic devices more frequently at mealtimes during the pandemic.

### 4.1. Changes in Eating Behaviors during the Pandemic

Most families changed their eating habits in some way during the pandemic, with many sharing that they were eating less healthily. This message is consistent with recent survey research in which families indicated unhealthy changes in eating habits during the first couple of months of the pandemic [26,27]. In the present study, focus group discussions allowed families to elaborate on changes in eating habits, which spanned changes in many areas, including the types of foods consumed and the amount. The types of food available at home, as well as the quantity, are powerful influences on children’s eating habits [17]. While healthy changes in eating habits are not possible for all families due to limited resources, for some families, strategies for healthier living during a pandemic may be welcomed. Some families in this study shared that they had intentionally made healthy eating changes during the pandemic. In similar situations, unified and consistent public health messages should be more strongly centered around encouraging families to eat healthier.

A recent study [24] found that child snacking increased in the first couple of months of the pandemic, and our study extends this research by showing that increased child snacking behavior persisted 9–11 months after the pandemic began, which may have lasting implications for child weight status. Additionally, in the present study, parents shared how children’s snacking influenced their own snacking behaviors. According to a family systems perspective, family members influence one another and changes in one subsystem can affect another subsystem [34]. Parents talked about how they would find themselves snacking along with their children, especially as they were preparing snacks and handing them out. Changes in some eating behaviors may persist even post pandemic, but a resumption of normal routines may shift behaviors to pre-pandemic levels. It can be expected that once children are back in school and following their usual routines that snacking behaviors will decrease to some extent just by virtue of being away from the home environment. While these decreases in snacking may be observed, excess weight gain will likely still need to be addressed. A recent review of 15 studies on obesity in children during the pandemic found support for an increase in child weight, which was associated with changes in eating habits and limited physical activity [35]. For parents, who may find themselves in work situations that combine office and remote work, changing snacking behaviors may be more challenging. Perhaps a comprehensive and integrated approach that includes healthcare professionals, schools, and parents working together can be effective at helping families to get back on track with resuming healthy habits and losing excess weight. This should be of paramount concern for public health [36], given the high rates of obesity in the nation [37] and the associated risks of hospitalization and morbidity linked to COVID-19 [38].

### 4.2. Positivity in Time Together

A chronic stressor such as an unprecedented pandemic, with cyclical lockdown restrictions and high unpredictability, has resulted in families spending more time together, confined to smaller spaces. This type of a situation could lead to higher rates of stress and irritability within families, but parents in this study shared that the increase in family time, particularly around mealtimes, has been positive overall. Mealtimes seemed to provide a central place for families to feel connected during an uncertain time. Research suggests that mealtimes are a setting in which difficult conversations can take place [39], which may be especially helpful during times of stress. Parents shared that this was a good opportunity to check in with one another, as well as to enjoy one another’s company. Family mealtimes have been associated with many benefits in the literature, and, during the pandemic, they seemed to offer solace for families. Intervention studies have been effective at increasing the frequency of family meals [14] and, for many families, the COVID-19 pandemic presented a natural way of increasing mealtime frequency. Future research should assess how the family meal can be used to leverage family wellness during future times of stress.

### 4.3. Screen Time during Meals

Device usage during mealtimes is associated with an increased risk of obesity [40,41]. TV viewing, specifically during meals is related to less healthy eating [40,42] and, in our study, TV viewing intensified during the pandemic. Whereas TV viewing during mealtimes was high pre pandemic, the pandemic created conditions in which TV viewing became even more exaggerated. Other electronics were also more likely to accompany mealtimes, including cell phones and video games. This type of behavior may be more resistant to change post pandemic. Additionally, the gradual nature of the resumption of normal activities during the pandemic may add to the difficulty of making lasting changes around screen time. Families may want to set new rules regarding screen time when routines are beginning to be reestablished in order to return to pre-pandemic screen-time levels. While studies have found that device usage during meals can interfere with family communication (e.g., [39]), parents in the present study shared that the time together during mealtimes was often spent in conversation and, overall, was positive.

### 4.4. Strengths and Limitations

The results of this study should be interpreted with caution. Families were all from California and all children were attending school remotely at the time of the study. There is wide variation across the nation with regard to COVID-19 restrictions, in-person schooling, and remote work. In addition, we did not focus on a specific age range, which limits the understanding of how the pandemic is influencing children by developmental stage. However, the goal of our study was to understand the family as an entity, and sampling families with children across age ranges allowed for a glimpse into the reality that many families are experiencing. While we actively recruited both mothers and fathers, only one father participated in the study. Mothers tend to be the decision-makers in the family food environment, and this study highlighted mothers’ roles during the pandemic, including their influence in shaping children’s health. Additionally, recent research found that mothers were more likely to report improvements in relationships with their children during the pandemic than fathers [43], which may be a reflection of the greater amount of time spent with them. Future research may want to specifically examine fathers’ perspectives during the pandemic, as research points to the role fathers also play in shaping children’s health behaviors around food (e.g., see [44,45]). Similarly, a study that combines parent and child perspectives may provide additional useful information about how children perceive changes in the food environment during chronic stressful periods and how those align with parent perspectives. Strengths include a predominantly Hispanic, SES diverse sample, which, to our knowledge, has been underrepresented so far in the emerging research on the COVID-19 pandemic’s effects on family functioning. Future research may want to include a focus on how families maintain connectedness through the frequency and quality of family meals post pandemic.

## 5. Conclusions

The combination of unhealthy eating, sedentary behaviors, and screen time during the pandemic has the potential to create lasting health effects. Research suggests that regular family meals are associated with healthier eating and a reduced risk of obesity [46], but overeating, excessive snacking, and screens may threaten to override positive effects. As the country begins to transition to a new normal, a return to healthier eating habits should be the focus of public health efforts. Future research should focus on mitigating potential lasting physical health effects from overeating and sedentary behaviors during the pandemic, as well as creating sustainable healthy habits that can be extended to similar situations in the future.

## Figures and Tables

**Table 1:** Participant characteristics. Income is listed in USD.

Demographics	*N* (%)
**Ethnicity**	
Asian	2 (4)
Hispanic	39 (81)
White	7 (15)
Median child age	11.5
**Education**	
Less than high school	14 (29)
High school	14 (29)
Technical school	4 (8)
Professional degree	2 (4)
Bachelor’s degree	5 (10)
Master’s degree	7 (15)
Declined to respond	2 (4)
**Income (annually)**	
$19,999 or less	13 (27)
$20,000–$29,999	5 (10)
$30,000–$39,999	6 (13)
$40,000–$49,999	5 (10)
$50,000–$59,999	3 (6)
$60,000–$69,999	3 (6)
$70,000–$79,999	2 (4)
$80,000–$89,999	1 (2)
$90,000–$99,999	1 (2)
$100,000 or more	8 (17)
Declined to respond	1 (2)

## Data Availability

Data are not available due to assurances of confidentiality.
